# Forgotten Fibrocytes: A Neglected, Supporting Cell Type of the Cochlea With the Potential to be an Alternative Therapeutic Target in Hearing Loss

**DOI:** 10.3389/fncel.2019.00532

**Published:** 2019-12-06

**Authors:** David N. Furness

**Affiliations:** School of Life Sciences, Keele University, Keele, United Kingdom

**Keywords:** cochlear fibrocyte, cochlear lateral wall, metabolic hearing loss, Ménière’s disease, cell replacement therapy

## Abstract

Cochlear fibrocytes are a homeostatic supporting cell type embedded in the vascularized extracellular matrix of the spiral ligament, within the lateral wall. Here, they participate in the connective tissue syncytium that enables potassium recirculation into the *scala media* to take place and ensures development of the endolymphatic potential that helps drive current into hair cells during acoustic stimulation. They have also been implicated in inflammatory responses in the cochlea. Some fibrocytes interact closely with the capillaries of the vasculature in a way which suggests potential involvement, together with the *stria vascularis*, also in the blood-labyrinth barrier. Several lines of evidence suggests that pathology of the fibrocytes, along with other degenerative changes in this region, contribute to metabolic hearing loss (MHL) during aging that is becoming recognized as distinct from, and potentially a precursor for, sensorineural hearing loss (SNHL). This pathology may underlie a significant proportion of cases of presbycusis. Some evidence points also to an association between fibrocyte degeneration and Ménière’s disease (MD). Fibrocytes are mesenchymal; this characteristic, and their location, make them amenable to potential cell therapy in the form of cell replacement or genetic modification to arrest the process of degeneration that leads to MHL. This review explores the properties and roles of this neglected cell type and suggests potential therapeutic approaches, such as cell transplantation or genetic engineering of fibrocytes, which could be used to prevent this form of presbycusis or provide a therapeutic avenue for MD.

## Introduction

The major tissues of the cochlea are notable for their discrete functional roles and the diversity of cell types that each possesses. At the same time, these tissues display a complex interdependence in which their disruption, either singly or in different combinations, leads to hearing impairment. There are three main tissues: the sensory epithelium (organ of Corti), located on the basilar membrane, that detects and transduces sound energy into an electrical response; the neural component, i.e., auditory nerve fibers and spiral ganglion, that conveys the responses of the organ of Corti along the auditory pathway; and the lateral wall, a homeostatic tissue that maintains electrochemical conditions within the cochlea favorable to the optimal function, and potentially survival of the sensory organ and the neural elements. In addition to these, there are also other tissue components whose functions are probably less directly involved in hearing, such as the endolymphatic sac and various periosteal linings (see for example Standring, 2015).

The diversity of functions and cell types present in these tissues means that there is a range of ways that degeneration affecting different targets contributes to hearing loss. The purpose of this review is to raise the profile of one cell type whose importance has been underestimated and contribution often neglected: the fibrocytes of the spiral ligament. A simple search of PubMed using the search terms “cochlear hair cell” and “cochlear fibrocyte” makes this neglect clear as the former (at the time of writing) obtained 11,557 hits whilst the latter obtained 262, a ratio of 44:1. On the basis of scientific endeavor, then, this might imply that fibrocytes contribute very little to cochlear function. On the basis of human pathological studies (Schuknecht and Gacek, 1993), however, the reality is that in fact, a significant proportion of hearing loss cases are likely to have either directly or indirectly a major contribution from lateral wall, and likely fibrocyte, dysfunction.

## The Different Types of Hearing Loss

Five-hundred million people worldwide have some form of hearing loss. Hearing loss is often subdivided into sensorineural (SNHL) and conductive (CHL; Sheffield and Smith, 2018). Around 90% is generally attributed to inner ear damage of some type, due primarily to aging, ototoxic drugs (chemotherapy/aminoglycoside antibiotics) and loud noises (e.g., soldiers, miners) and using the definition above, this would thus be considered SNHL where the sensory hair cells in the organ of Corti and/or spiral ganglion nerve (SGN) cells that form the auditory nerve are affected. SNHL also includes the recently described “hidden hearing loss” resulting from synaptopathy at the level of auditory nerve input (Viana et al., 2015). The problem with this definition, however, is that hearing loss can also result from cellular degeneration in the cochlea in the other tissues noted above, sometimes separately, sometimes together. Thus, some definitions include metabolic hearing loss (MHL), where lateral wall structures (spiral ligament and *stria vascularis*) are affected as distinct from SNHL (Schuknecht and Gacek, 1993; Dubno et al., 2013). Currently, no cure exists either for MHL or SNHL, though hearing loss, in general, is managed by prosthetics (hearing aids and cochlear implants).

The relative proportion of MHL and SNHL in age-related hearing loss patients, and with what frequency both may be present at some point in the patient’s disease progression, is uncertain. Because SNHL is often quoted as the major cause of hearing loss, hair cell repair or regeneration is the commonest target of most research in cochlear therapies. However, the pathological studies of human temporal bones by Schuknecht and Gacek (1993) suggest that the greater proportion of such acquired hearing loss could be MHL on the basis of a pattern of lateral wall atrophy. Whilst this is likely to be contentious, if correct, this would change our general perception of hearing loss. As it stands, however, there is insufficient evidence to be certain, so this needs to be confirmed.

The lack of data on the relative proportion of MHL compared with SNHL is presumably because there are no good diagnostic techniques to distinguish clearly between these disorders. Such techniques are being developed on the basis of animal models (Dubno et al., 2013; Vaden et al., 2017, 2018) and are likely to be in place in the relatively near future. More refined diagnostic techniques would enable the clinician to determine, with greater accuracy than presently possible, the form of the hearing loss and therefore identify patients who would be amenable to appropriately targeted treatment.

## The Structure and Fluid Composition of the Cochlear Duct

**Figure 1:**
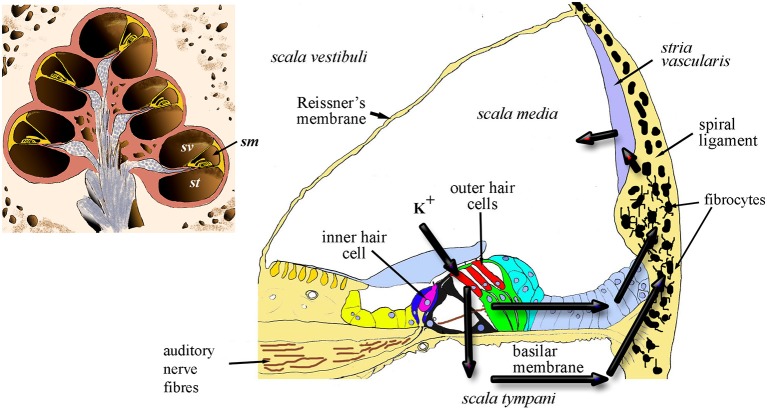
Diagrammatic representation of the cochlea in section (inset) showing the chambers of the spiral and the location of *scala media* (sm) *scala vestibuli* (sv) and *scala tympani* (st). The main panel shows a cross-section of the sm and the arrows indicate the two probable routes of recycling potassium through the organ of Corti, perilymph and lateral wall, including the fibrocytes.

To understand the concept of lateral wall-based MHL, it is necessary to review briefly the structure of the cochlea and cochlear homeostasis, with an emphasis on the region of the spiral ligament. The spiral cochlea contains the sensory organ of Corti located in the cochlear duct which is the middle chamber of three fluid-filled chambers (Figure 1) that run longitudinally along the cochlea. These are the *scala vestibuli* and *scala tympani*, which are in continuity at the cochlear apex and are filled with perilymph, a solution high in sodium ions and containing a number of other ions and substances, and the *scala media* that contains endolymph which is high in potassium ions. The *scala media* is separated from the other two compartments by a lining of epithelial cells of various types, all of which are connected by tight junctions that prevent paracellular translocation of ions and other substances, thus forming a complete electrochemical barrier between the inside of the *scala media* and the surrounding tissues and extracellular spaces.

The tight junction barrier on the inside of the *scala media* lining ensures that the composition of cochlear fluids is under the control of the cells lining the epithelium, in the sense that the only access between the inside of the duct and the outside is through the plasma membranes of the cells facing the inside. Hence the transfer of ions or substances of any kind is limited to apical membrane channels or transporters on these cells. Maintenance of the correct fluid composition in the different compartments is thus dependent on each cell type contributing to the re-distribution of ions in an appropriate manner. This is vital for normal auditory function, specifically in providing support for the function of the sensory hair cells (see review by Wangemann, 2006).

The tight junctions in the organ of Corti occur between the adjoining apical surfaces of hair cells and supporting cells, which form a region called the reticular lamina. Each hair cell is characterized by a sensory hair bundle that projects into the endolymph in the *scala media*, whilst the cell bodies below the reticular lamina are bathed in perilymph. During the sensory functioning of the hair cells, the only route for transfer of ions across this surface is through channels located in the hair bundles; stimulation by sound causes these mechanotransducer channels to open resulting in entry predominantly of K^+^, but also Ca^2+^, which depolarizes the hair cells, resulting in the release of the neurotransmitter glutamate at synapses between auditory nerve fibers and the basal pole of the hair cells. This, in turn, leads to stimulation of spiral ganglion neurons (SGNs) which then signal the hair-cell response to the brain. Potassium ions exit the hair cells into perilymph *via* basolateral potassium channels (see review by Wangemann, 2006). To our knowledge, the supporting cell apical surfaces do not express ion channels or transporters, but the cells are connected sub-apically by gap junctions which help to form an epithelial gap junction network as described below.

In essence, continued active redistribution of the K^+^ ions results in a powerful battery, the endocochlear (or endolymphatic) potential (EP) that drives current into the hair cells whilst their transduction channels are open. Exhaustion of K^+^ ions would reduce the magnitude of transduction; thus loss of EP results in loss of auditory nerve function (Lang et al., 2010). In addition, excessive K^+^ in hair cells appears to result in hair-cell death (Nouvian et al., 2003). This toxicity can be avoided only because potassium is continuously pumped out of the perilymph. Failure to maintain the correct balance of fluids within these compartments might also lead to volumetric changes that result in endolymphatic hydrops, a likely origin of Ménière’s disease (MD). The lateral wall tissue is of prime importance in this process by contributing to two major routes for recycling the K^+^ ions released into perilymph from the hair cells (Figure 1). These routes involve transcellular relocation of the K^+^ ions through the ligament. The source of the ions is either directly from perilymph or *via* the gap-junction connected supporting cells of the organ of Corti. The breakdown of these homeostatic mechanisms can result in failure to recycle this crucial ion between the different fluid-filled compartments of the cochlea.

## Lateral Wall Structure and Function

The two major components of the lateral wall: the *stria vascularis* and the spiral ligament, function together to maintain the EP during K^+^ recycling. As shown in Figure 1, the recycling occurs *via* the two main routes from the perilymph around the hair cell bases. The first of these is a transcellular route (the epithelial gap-junction network) *via* the cells of the outer sulcus which are connected to each other by gap junctions composed of connexins such as Cx26, Cx30 and Cx31 (Xia et al., 1998; Forge et al., 1999; Mei et al., 2017). This is the epithelial gap junction network and the cells involved form an epithelial syncytium where their cytoplasmic compartments are in direct contact. Its importance is exemplified by the fact that genetic mutations in these genes are significant contributors to genetic hearing loss, for example, GJB2 (Cx26), GJB6 (Cx30) and GJB3 (Cx31; see for example Forge et al., 2003). Gap junctional proteins have also been confirmed directly in histological studies to occur in the human spiral ligament (Liu et al., 2017).

The second route for potassium transfer is through perilymph. This also ends up at the lateral wall where potassium ions enter the lateral wall gap-junction system (the connective tissue gap junction network or syncitium).

**Figure 2:**
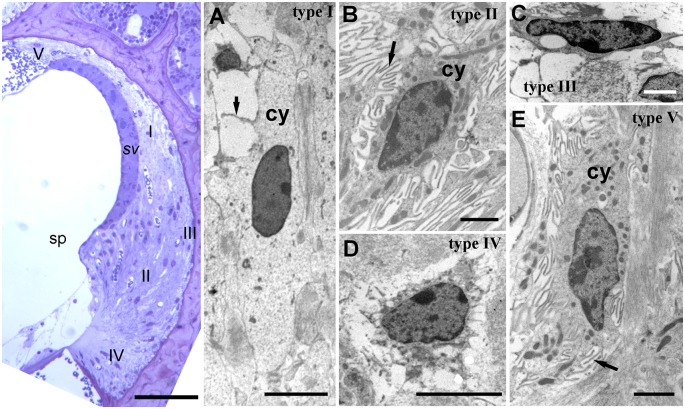
Lateral wall structure in a CD/1 mouse. The left panel shows a light microscopy section of *stria vascularis* (sv) spiral prominence (sp) and the spiral ligament containing five types of fibrocyte (I—V). Scale bar = 50 μm. Panels **(A–E)** show fibrocyte types I to V by transmission electron microscopy. They have varying numbers of small processes (arrows) and differences in the density of cytoplasm (cy) and organelle content. Scale bars: **(A,C)** = 5 μm; **(B,D,E)** = 2 μm (adapted with permission from Mahendrasingam et al., 2011a).

The *stria vascularis* is a heavily vascularized three-cell layered tissue composed of marginal cells facing the *scala media*, intermediate cells and basal cells. This tissue lines the inner curved surface of the spiral ligament between a spiral ridge of tissue (the spiral prominence) and the junction of the ligament with the upper boundary of the cochlear duct, Reissner’s membrane. The ligament itself is longer, extending above and below the *stria vascularis*; at its lower end is the anchor point of the basilar membrane, whilst extending above Reissner’s membrane it lines the bony external wall to the upper boundary of the spiral chamber (Figure 2). The ligament varies in width in different cochlear turns, becoming wider towards the basal end of the cochlea. Cell types and densities thus vary along the cochlear duct, but quantitative information about these changes is, thus far, limited.

The cell types of the ligament include perivascular endothelial cells around capillaries, root cells (a large, branched cell type projecting from near the basilar membrane anchorage and midway into the ligament) and fibrocytes. There are five main types of fibrocyte in mice, with differential morphology, protein expression and location (Figure 2; Furness et al., 2009; Mahendrasingam et al., 2011a,b) although in other studies, for example in gerbil, these have been divided into several further subtypes (Spicer and Schulte, 1996).

From electron microscopic studies, the fibrocytes are distinguished by fine structural characteristics such as the density of cytoplasm, content of cell organelles and the extent to which the plasma membrane is folded (Figure 2). Thus, the least complex of the cells structurally is the type I fibrocyte which has an elongated shape, relatively light cytoplasm and few organelles, and a minimal number of plasma membrane folds. Type II and type V are structurally very similar to each other, have relatively dense cytoplasm and multiple membrane folds. Type III tend to have elaborately branched surfaces, and very dense cytoplasm, with narrow cell bodies. Type IV cells are similar to but generally larger than type III with intermediate amounts of surface elaborations between type III and type II or type V.

The role of these various fibrocyte types is uncertain, but they show other differences in characteristics in terms of protein expression (Table 1). As with the epithelial syncitium, the gap junction network of the ligament comprises several connexins (Cx26, Cx30, Cx31 and Cx43). It is known that type I fibrocytes are connected by gap junctions to the basal cells of the *stria vascularis* (Forge et al., 2003), and that type II and type V cells possess high levels of the Na, K, ATPase transporter, whilst type I cells have less and type III and type IV cells have little (Mahendrasingam et al., 2011a). To some extent, these distributions have been confirmed in human tissues as well (Liu et al., 2017), although type I cells were not found to express the NaKATPase. This perhaps reflects age or sampling issues of human tissues. Type II cells appear to be connected to type I cells in the gap junction network. Other proteins expressed in fibrocytes include the potassium channel Kir5.1 (Hibino et al., 2004), BK channels (Liang et al., 2003; Shen et al., 2004), L-type Ca2^+^ channel (Liang et al., 2004) and Na-K-Cl co-transporter 1 (NKCC1; Crouch et al., 1997) which are likely to be involved in the recycling of K^+^. Type III cells (alternatively called tension fibroblasts) contain prominent cables of actin filaments and may modulate tension between the basilar membrane and the bony wall of the cochlea (Henson and Henson, 1988), but they also, uniquely amongst fibrocytes of the cochlea, express aquaporin 1, implicating them in water homeostasis (Mahendrasingam et al., 2011a).

**Table 1:** Characteristic proteins of fibrocytes.

Protein	Function	Strongly expressing	References
Caldesmon	Calcium modulation	Type III	Mahendrasingam et al. (2011a)
S-100	Calcium modulation	Type I, II and V	Suko et al. (2000) and Mahendrasingam et al. (2011a)
Na, K-ATPase	Sodium/potassium transport	Type II and V	Suko et al. (2000) and Mahendrasingam et al. (2011a)
Ca, ATPase	Calcium transport	Type I	Ichimiya et al. (1994)
Na, K, Cl-cotransporter	Sodium/potassium/chloride transport	Type II, IV and V	Qu et al. (2006)
NCBE	Bicarbonate transporter		Huebner et al. (2019)
GLAST	Glutamate transport		Jin et al. (2003) and Furness et al. (2009)
Kir5.1	Potassium channel	Type II, IV, and V	Hibino et al. (2004)
Ether-a-gogo	Potassium channel		Nie et al. (2005)
Kv3.1	Potassium channel		So et al. (2001)
BK channels	Potassium channel		Liang et al. (2003)
ClC	Chloride channels		Qu et al. (2006)
Connexin 26, 30, 31, 43	Gap junction channel		Forge et al. (2003)
AQP1	Water channel	Type III	Miyabe et al. (2002) and Mahendrasingam et al. (2011a)
Carbonic anhydrase	Carbon dioxide-water converter	Type I, III, IV and V	Spicer and Schulte (1991)
Creatine kinase	ADP-ATP cycling	Type I, III, IV and V.	Spicer and Schulte (1991)
Connective tissue growth factor (CTGF)	Collagen metabolism	Type IV	Adams (2009)

*Table illustrating some of the proteins known to be of functional relevance in fibrocytes and often used to characterize the different types*.

Root cells have extensive broad processes penetrating into the lower part of the ligament. They also have gap junctions communicating with the surrounding cells and are supported by a tubulin/microtubular cytoskeleton, presumably to maintain the extensive branches (Jagger et al., 2010).

**Figure 3:**
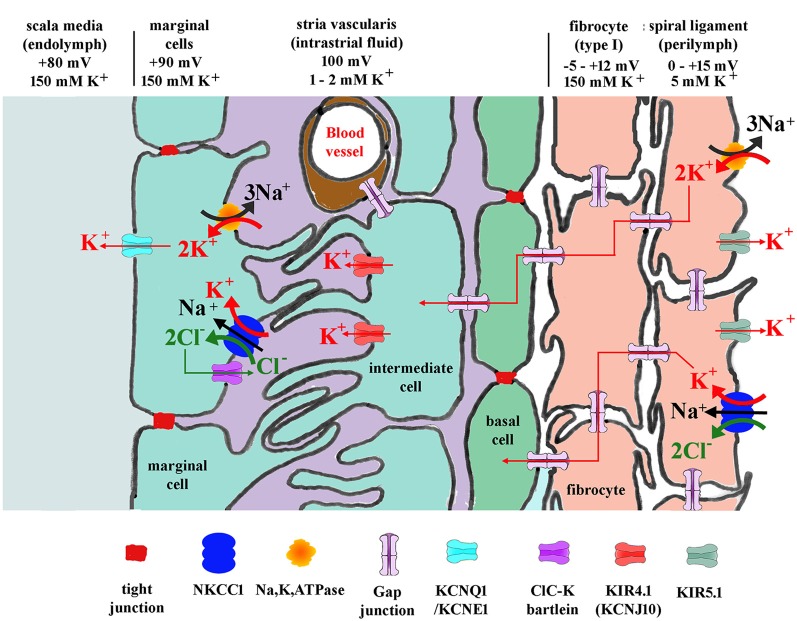
Diagram showing potassium and other ion circulation and the formation of the EP within the lateral wall. In the stria vascularis and the spiral ligament, K^+^ concentration gradients ([K^+^]) and potentials vary at different points in the tissue as shown. Gap junctions connect the spiral ligament fibrocytes together and also connect them to basal cells and then intermediate cells of the stria vascularis. K^+^ entering from outer sulcus cells of the organ of Corti or directly from perilymph *via* Na, K, ATPase or NKCC1 then flows through the syncytium of the ligament into the stria vascularis where it is transported to marginal cells and secreted into endolymph. For protein identity, see key below diagram. Modified figure based on Hibino and Kurachi (2006) with additional data from Adachi et al. (2013).

The homeostatic K^+^ recycling mechanism involving fibrocytes is generally presumed necessary to ensure maximum sensitivity of the sensory hair cells (Wangemann, 2006) and also to maintain the conditions which ensure the continued survival of other cochlear tissues. The K^+^ then travels to the *stria vascularis* basal cells lying adjacent to the spiral ligament. *Stria vascularis* function is perhaps better understood than that of the ligament. The molecular players involved in the redistribution of ions suggest a kidney like function, with concentration gradients that drive the relocation of potassium. This is thought to be initiated by transport into the basal cells *via* the gap junction connections with type I fibrocytes (Figure 3). The *stria vascularis* is also compartmentalized from the ligament by tight junctions composed of claudin 11 in both humans and rodents (Kitajiri et al., 2004; Liu et al., 2017), ensuring that cellular ion transport mechanisms are the route for K^+^.

## Physiological Properties of Fibrocytes

Electrophysiological studies of fibrocytes have been fairly limited, but there is evidence that they have unusual properties that may be associated with a potassium recycling role. Typically eukaryotic cells have a negative resting membrane potential, but fibrocytes have been reported to possess positive potentials *in vivo* of up to +12 mV (Yoshida et al., 2016). It has been suggested that this contributes to the establishment of appropriate ionic gradients inside the lateral wall that enable the development of the EP through the *stria vascularis*. Studies of the potentials within the ligament suggest it is slightly positive compared with perilymph. Blockers of Na, K, ATPase decrease K^+^ concentration within the fibrocytic zone and reduce EP (Adachi et al., 2013) providing evidence for the role of fibrocytes in maintaining EP.

Our study of glutamate transport properties of fibrocytes in slices from rat pups (Furness et al., 2009) established that the cells had a glutamate transporter-associated current that corresponded with the amount of GLAST in them—i.e., type II fibrocytes which express GLAST more than other fibrocytes had greater glutamate transport capability than type I fibrocytes which express less GLAST. Since GLAST is a co-transporter of Na^+^ and K^+^, it may also contribute to the recirculation of K^+^. However, during glutamate uptake, K^+^ is transported out of the cytoplasm—thus GLAST would in principle be operating in an opposing direction to the Na, K, ATPase with respect to K^+^.

Other physiological properties that have been directly measured have been noted in *in vitro* studies of fibrocyte cultures. These suggest that fibrocytes also possess chloride channels (Qu et al., 2006).

## A Role for Fibrocytes in Inflammatory Responses

Cochlear inflammation is often triggered by middle ear infection or damage caused by environmental insults such as acoustic trauma or ototoxic drugs. Fibrocytes are thought to play a role in inflammatory responses in the cochlea and to offer a potential for protection from inflammation that could lead on to SNHL. In experimental models of inflammatory triggers, immunostaining for Na, K, ATPase and Cx26 have been shown to be decreased in labyrinthitis while in otitis media Cx26 is reduced (Ichimiya et al., 2000). The same authors showed that cultured spiral ligament fibrocytes release chemokines after stimulation by proinflammatory cytokines, TNF-α or IL-1β. IL-10 mediated protection from ototoxic drugs has also been observed (So et al., 2001), suggested to be associated with an anti-inflammatory response in fibrocytes (Woo et al., 2015). Hence it is likely that as part of their homeostatic role, the fibrocytes have anti-inflammatory capabilities that have the potential to protect the cochlea from inflammation leading to deafness.

## Lateral Wall and the Blood-Labyrinth Barrier

As well as K^+^ recycling, the lateral wall is a major contributor to the blood supply of the cochlea. In this context, the functional relationship of lateral wall fibrocytes to the blood-labyrinth barrier is of potentially great interest, but as yet is unclear. It is likely that the *stria vascularis* is the main site of the blood-labyrinth barrier, as indicated by its heavy vascularization. It is perhaps less well known that the spiral ligament is well vascularized (Carraro et al., 2016). Indeed, ultrastructural studies suggest a close association between some fibrocyte types and the blood vessels of the ligament (Spicer and Schulte, 1996) including endfeet-like processes (Dai and Shi, 2011), where material transport between blood and labyrinth tissue is likely to be tightly controlled. Furthermore, tracer studies showed that spiral ligament vessels have lower permeability than those of *stria vascularis*, with tight junctional barriers between the endothelial cells of the blood vessels (Sakagami et al., 1982). How this influences the homeostatic activities and barrier properties of the lateral wall remains to be established.

More indirectly, a role for fibrocytes in vascular control of the cochlear blood supply has been suggested. The study by Dai and Shi (2011) found elevation of Ca^2+^ in fibrocytes generates a Ca^2+^ signal to nearby vascular cells causing vasodilation of capillaries. This coupling was also found to mediate sound-stimulated cochlear blood flow increases.

## Lateral Wall Pathology in Hearing Loss

Evidence from both animal and human deafness studies suggests that degeneration of the lateral wall fibrocytes is associated with hearing loss and a contributor probably to MHL. Time course studies suggest that fibrocyte degeneration can precede hair cell loss (Hequembourg and Liberman, 2001; Mahendrasingam et al., 2011b), so it is possible to surmise that it could also lead to SNHL by giving rise to a change in the composition of cochlear fluids that negatively affect hair cells and neural elements. One human deafness gene (DFN3) has been identified that has degeneration of fibrocytes as its major pathology, accompanied by severe reduction of the EP in an animal model (Minowa et al., 1999), and fibrocyte degeneration has been reported in mice showing age-related hearing loss (Hequembourg and Liberman, 2001). We found early fibrocyte pathology, including damaged and degenerating cells, in CD/1 mice that show accelerated age-related hearing loss (Mahendrasingam et al., 2011b). The degeneration was primarily, in its initial stages, in the form of mitochondrial damage, as is found in other types of age-related degeneration (see review by Zhu et al., 2019). These findings led us to hypothesize that fibrocyte degeneration is a significant cause of MHL leading to SNHL.

In their study of human temporal bones of a range of ages, Kusunoki et al. (2004) found significant early loss of types II and IV fibrocytes followed by types I and III, respectively. From the ultrastructural studies noted above Spicer and Schulte (1996), loss of type II cells and their association with blood vessels might well compromise the blood-labyrinth barrier in the type II location. This could lead to disturbances of cochlear fluid composition, increased vulnerability to toxins, and deleterious changes leading to further pathological alterations, potentially loss of EP and hearing loss.

It should be noted, however, that it is not always clear that loss of EP produces hearing loss. Although measurements of EP in some cases of threshold elevation show EP losses (Mei et al., 2017) others do not (Lukashkina et al., 2017). This lack of consistency in data from the literature makes it harder to make the case for MHL through *stria* or ligament dysfunction. Nevertheless, the temporal bone studies showing that lateral wall pathology is seen with deafness in older people remains a good indicator that there is a link.

## Lateral Wall and Ménière’s Disease

As well as hearing loss, lateral wall pathology has been implicated in MD. Whilst some of the symptoms of MD can be attributed to a loss of the sensory hair cells in the inner ear, other pathological changes have also been noted in endolymphatic hydrops, a feature of MD. In a study of experimental (surgically induced) endolymphatic hydrops in guinea pig, Nadol et al. (1995) evaluated pathological changes throughout the cochlea over a period of time up to 6 months. They found that type I fibrocytes showed early changes with downregulation of expression of a variety of proteins tested (e.g., S-100, Ca, ATPases and other enzymes) and later type I and type II fibrocytes showed reduction in Na, K, ATPases and connexin 26, along with structural degeneration. This led Merchant et al. (2005) to evaluate the relationship between endolymphatic hydrops and MD in a human temporal bone archive. They found hydrops in all MD cases, although not always MD in hydrops cases, and concluded that spiral ligament pathology was clearly involved with both hydrops and MD.

**Figure 4:**
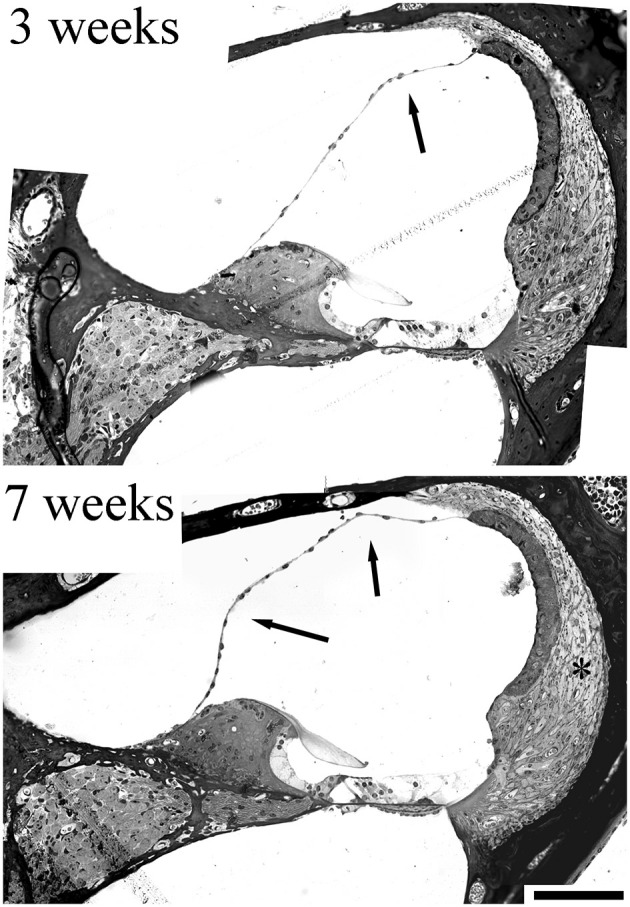
Sections from a 3 week and 7 week CD/1 mouse cochlea showing accelerated age-related hearing loss. Fibrocyte degeneration in these mice has been reported (Mahendrasingam et al., 2011b) and can be seen as loss of cell nuclei/thinning of the tissue (*). The evidence for progressive hydrops is indicated by the arrows, where Reissner’s membrane is distorted at 3 weeks, but this distortion becomes more exaggerated at 7 weeks, implying an increase in volume. Scale bar = 50 μm (adapted with permission from Mahendrasingam et al., 2011b).

An evaluation of the CD/1 mice in our studies (Mahendrasingam et al., 2011b) also hinted at a link between MD and fibrocyte pathology. Although we did not report the pathology at the time, it is evident that sections of the cochlea show a distended cochlear duct, with Reissner’s membrane being deformed (Figure 4) to an increasing extent with age, a possible indicator of MD. Although this distortion was not always present in older CD/1 mice, it might suggest that fibrocyte degeneration contributes to fluid changes in the cochlea.

Aquaporin 4 and 6 (proteins involved in water homeostasis) expression also seem to be affected in MD (Ishiyama et al., 2015) although aquaporin 1 (expressed by type III fibrocytes) was not reported to be affected. Nevertheless, degeneration of type III fibrocytes seems likely to occur along with the other fibrocytes, as we have seen in our studies of CD/1 mouse lateral wall degeneration, and this would cause loss of aquaporin 1 and potentially osmotic changes. The role of aquaporin 1, therefore, needs further investigation. The distribution of the glutamate transporter GLAST, presumed to be involved in glutamate recycling, is altered in the lateral wall in Ménière’s patients (Ahmed et al., 2013). Build-up of glutamate in perilymph could potentially lead to excitotoxicity in the cochlea, affecting the SGNs nerve terminals with the hair cells; indeed glutamate based excitotoxicty of SGN terminals on hair cells has been demonstrated in a number of studies (see review by Pujol and Puel, 1999). Finally, Ishiyama et al. (2007) found a significant reduction against age-matched controls in both *stria* and spiral ligament volume in five temporal bones from humans suffering from MD.

Thus, although a direct, causal link between any specific pathology and MD has yet to be established, there is good evidence that fibrocyte degeneration is highly likely to be a contributory factor.

## Fibrocytes as a Potential Target for Therapy in Hearing Loss and MD

The lateral wall of the cochlea is relatively superficial as a structure, compared with hair cells or SGN. Substances could potentially be introduced into this area through the round window or an opening in the cochlear bony wall. Its superficial location also means it is more accessible than deeper structures for surgical intervention. The tough ligament itself is likely to be better able to withstand surgical procedures or physical manipulation than other more delicate tissues in the cochlea, although with the proviso that disruption of the blood-labyrinth barrier in the ligament, or of the tight junction network on the inner surface of the *scala media*, might prove damaging. It has proved possible to make a small opening in the bony wall of the guinea pig cochlea through which electrodes can be introduced into the ligament and *stria vascularis* (Yoshida et al., 2016). The question then is what might be introduced into the ligament that could assist in the repair and restoration of homeostasis, or prevention of hearing loss?

Intervention in spiral ligament degeneration could take several forms: (i) stimulation of the fibrocytes’ natural proliferative capacity to increase repair potential; (ii) alteration of the physiology of the cells to enhance their homeostatic activities, e.g., through transfection with genes to overexpress functional proteins, or even to express new proteins that might support other cells in the cochlea (e.g., growth factors); and (iii) direct replacement of fibrocytes through cellular transplantation. These options require a better knowledge of the functions of fibrocytes and ways to manipulate them, as well as a source of fibrocytes *in vitro*. The latter provides not only an experimental platform in which to study them (see for example Qu et al., 2006) but also a possible source of cells for transplantation therapy.

**Figure 5:**
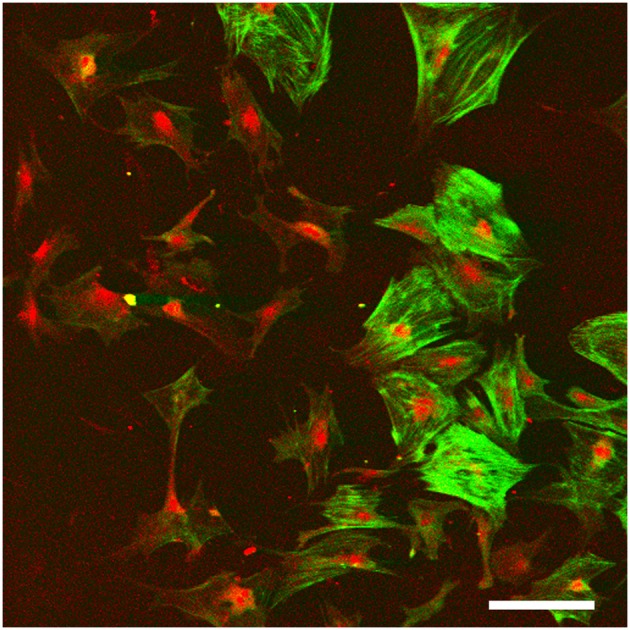
Monolayer fibrocyte culture after immunofluorescence labeling for caldesmon (green) and Na, K, ATPase (red). The cells exhibit a mixed phenotype with some cells labeled strongly for both, and others labeled primarily for the Na, K, ATPase. Scale bar = 100 μm. Image taken from conference presentation, cited in published proceedings by Furness (2014).

Accordingly, stem cells that have the potential to form fibrocytes, or direct culture of cochlear fibrocytes are possible ways forward in achieving the options suggested above. The developmental germ layer of origin of fibrocytes is mesoderm, and as cochlear fibrocytes form part of a connective tissue, they are thus considered to be mesenchymal cells. Such cells commonly retain a proliferative capacity throughout life and can thus be stimulated to grow; a related cell type, circulating fibrocytes in blood, are involved in wound healing and scar formation, producing myofibroblasts in fibrotic lesions (Quan et al., 2006). However, although a small degree of proliferation has been detected within the lateral wall in adult cochlea (Li et al., 2017), this proliferative capacity seems to be insufficient to maintain fibrocyte populations or reverse the degenerative processes. One positive outcome though of this proliferative capacity is that once removed from the lateral wall it has proved possible to grow fibrocytes *in vitro* (Gratton et al., 1996; Suko et al., 2000; Figure 5). Alternatively, they could be derived from mesenchymal stem cells (MSCs) that are repurposed into fibrocytes (Kamiya et al., 2007).

Fibrocyte monolayer cultures are obtained by extracting pieces of spiral ligament and placing them in culture wells with a coverslip over the top (Figure 5). After a few days, the cells appear in the well around the ligament pieces (Gratton et al., 1996) and these can then be passaged into sub-cultures. Characterization of these cultures at first suggested that they contained type I fibrocytes as they were negative for Na, K, ATPase but positive for other markers suggested being characteristic of type I cells (Gratton et al., 1996; Suko et al., 2000). However, such classification is difficult to verify as it relies on visual confirmation and is not quantitative. For example, semi-quantitative analysis has shown that type I fibrocytes do express moderate levels of Na, K, ATase relative to type III and type IV fibrocytes which express none, whilst type II and type V express the most (Mahendrasingam et al., 2011b). Other substrates are also possible, and 3D culturing of fibrocytes is another way forward. Preliminary work in our lab (Furness, 2014) has shown that fibrocytes can be grown on a variety of substrates and 3D culturing of type III fibrocytes from guinea pig in hydrogels has been successful (Kelly et al., 2012). This paves the way for novel strategies to grow fibrocytes and use of an appropriate matrix that could direct the phenotypes of the cultured cells, even to potentially “bioprinting” a replacement ligament *in vitro*.

The use of MSCs as an alternative to cultured fibrocytes also has some evidence to support it. It has been shown in one mouse model where the cochlear lateral wall was damaged by a mitochondrial toxin, that transplantation of MSCs by injection into the semi-circular canal located near to the cochlea produced some recovery of hearing function after loss (Kamiya et al., 2007). In this study, the MSCs were postulated to become fibrocytes once they had been integrated into the lateral wall and other regions containing fibrocytes (e.g., the spiral limbus running along the organ of Corti—Figure 1). Although there was no ultrastructural evidence that these transplanted MSCs had become fibrocytes, nevertheless they were in the correct locations and expressed connexin 26 and 30 as found in native fibrocytes. Whilst the mitochondrial toxin approach used in their study is not a natural degenerative process, nevertheless a hallmark of the degeneration we have observed is mitochondrial damage (Mahendrasingam et al., 2011b), hence giving confidence to the notion that a cell-replacement therapy for fibrocytes is a practical way forward.

Genetic engineering of fibrocytes is a relatively novel concept and there are a lot of unknowns as it is a strategy that has not been explored to any great extent. Cultures of fibrocytes can be used to test transfection strategies and develop either genetically engineered fibrocytes for transplantation or transfection methods that could be applied to native cells *in vivo*. For example, using cultured fibrocytes, we can test whether transfection will enable them to generate survival factors and release them. If genetically engineered cells are successfully incorporated into the lateral wall, they could be used to enhance the survival of other cell types in the cochlea (see below). Fibrocytes have been targeted in a very limited number of studies for transfection (only two to our knowledge: Zhuo et al., 2008; Oh et al., 2012).

Finally, it may also be possible to functionalize the fibrocytes in novel ways, e.g., by optogenetics to enable them to be more effective in potassium transport. A recently developed light-sensitive potassium channel shows that it may be possible to alter their potassium transport ability in response to light (Alberio et al., 2018). This could potentially be used to manipulate EP generation by stimulating fibrocytes through an external route *in vivo*. These approaches are clearly speculative, but there is much to be gained from exploring these potential new avenues.

## Other Advantages of Fibrocyte Manipulation

Arresting the degeneration of the lateral wall is likely to have other consequences for hearing function if the degeneration of hair cells and/or the spiral ganglion in SNHL is, to some extent, triggered by fibrocyte degeneration (Mahendrasingam et al., 2011b). Thus, lateral wall repair could potentially prevent subsequent SNHL. Furthermore, engineering fibrocytes to express certain growth factors could increase the longevity of the replacement fibrocytes and also be used to support long-term survival of both hair cells and supporting cells in the organ of Corti. Insulin-like growth factor 1 (IGF1) is thought to be a survival factor for hair cells and is currently undergoing clinical trials as a therapeutic agent (Yamamoto et al., 2014; Yamahara et al., 2015). Another growth factor that is potentially useful is brain-derived neurotrophic factor (BDNF) which helps to support SGNs (Leake et al., 2011). Survival of the SGNs is a requirement for the successful use of a cochlear implant long term after profound hearing loss caused by hair cell loss. This prosthetic device produces electrical pulses that stimulate SGNs and provides a signal that preserves some of the frequency content of the stimulus, replacing that derived from the missing hair cells, which normally are innervated by the neurons. The implant is amenable to working together with biological regenerative therapies (Roemer et al., 2017).

## Conclusions

The fibrocytes of the lateral wall of the cochlea are known to go missing in certain forms of hearing loss, and temporally, show early signs of loss of degeneration in humans and animal models. This leads to potential breakdown of cochlear homeostatic mechanisms and loss of the endocochlear potential. An under-recognized category of hearing loss—MHL—seems to be the likely consequence of this degeneration, and it may lead to SNHL in later stages. Fibrocytes thus constitute a relatively novel target in strategies to prevent hearing loss. Another positive attribute of these cells is that they are especially amenable to both investigation *in vitro* and enhancement or replacement *in vivo* because of their proliferative capacity which enables them to be grown in culture from adult cochleae. Therapeutic strategies involving fibrocytes thus hold great potential for the future.

## Author Contributions

DF conceived the topic area and wrote the review in its entirety.

## Conflict of Interest

The author declares that the research was conducted in the absence of any commercial or financial relationships that could be construed as a potential conflict of interest.
